# Bilateral occipital lobe infarction with altitudinal field loss following radiofrequency cardiac catheter ablation

**DOI:** 10.1186/1471-2261-10-14

**Published:** 2010-03-17

**Authors:** Susie T Luu, Andrew W Lee, Celia S Chen

**Affiliations:** 1 South Australian Institute of Ophthalmology, Discipline of Ophthalmology and Visual Sciences, University of Adelaide and Royal Adelaide Hospital, Adelaide, Australia; 2 Flinders Comprehensive Stroke Centre Medical Centre and Flinders University, Bedford Pk, Adelaide, South Australia, Australia; 3 Department of Ophthalmology, Flinders Medical Centre and Flinders University, Bedford Pk, Adelaide, South Australia, Australia

## Abstract

**Background:**

Bilateral stroke following radiofrequency catheter ablation is an unusual complication and may result in bilateral altitudinal visual field defects. Bilateral altitudinal visual field defects usually result from prechiasmal pathology causing damage to both retinas or optic nerves and rarely from bilateral symmetric damage to the post chiasmal visual pathways.

**Case presentation:**

A 48-year-old man complained of visual disturbance on wakening following radiofrequency catheter ablation. The patient had a CHADS score of 1 pre-operatively and no complications were noted intra-operatively. Examination revealed a bilateral superior altitudinal defect and MRI of the brain showed multifocal areas of infarction predominantly involving the occipital lobes which correlated to with the visual deficits.

**Conclusion:**

While the risk of thromboembolism and perioperative stroke during radiofrequency catheter ablation is small, it is not insignificant.

## Background

Radiofrequency catheter ablation is one method of restoring sinus rhythm in patients with atrial fibrillation (AF) [1]. It may be associated with serious adverse effects with a reported peri-operative risk of stroke of between 0% to 7% (median of 0.9%) [1,2]. Described us a first case of a bilateral altitudinal field defects secondary to bilateral occipital lobe infarction following radiofrequency cardiac catheter ablation.

Bilateral altitudinal field defects more commonly result from prechiasmal pathologies affecting both retinas or optic nerves rather than processes causing bilateral symmetric damage to the post-chiasmal visual pathways [3]. Bilateral occipital lobe infarcts, as seen in our case, can result in bilateral superior altitudinal field defects.

## Case presentation

A 48-year-old man underwent percutaneous cathether ablation for atrial fibrillation. Four years previously he had been diagnosed with a dilated cardiomyopathy after presenting with an episode of atrial fibrillation with an ejection fraction of 17%. He had no history of hypertension, diabetes or previous strokes. He was commenced on perindopril, metoprolol and warfarin. Initially, his atrial fibrillation was paroxysmal, however, over the last nine months he had noticed an increase in frequency of palpitations. Particularly in the last two months, he felt the atrial fibrillation was continuous and he was much less energetic and easily fatigued.

The patient had been anticoagulated with warfarin until 4 days prior to the procedure when the warfarin was withheld and anti-coagulation continued with subcutaneous low molecular weight heparin. A transoesophageal echocardiogram had been performed one week prior to the ablation procedure and showed no pre-formed atrial thrombus. The patient underwent segmental pulmonary vein isolation and linear ablation within the left atrium for substrate modification, as well as linear ablation of the cavo-tricuspid isthmus for ablation of the classic right atrial flutter circuit. An irrigated catheter tip was used. Intravenous heparin was commenced the morning of and during the procedure with a target activated clotting time of between 300 and 350 seconds.

**Figure 1:**
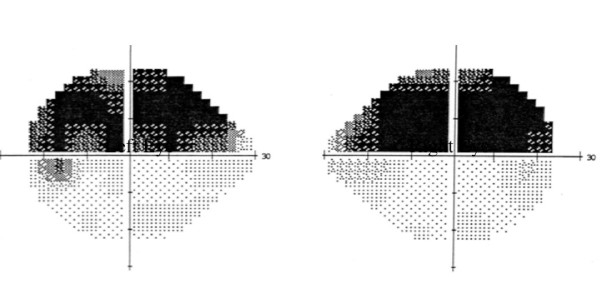
**Automated perimetry using Humphrey 30-2 threshold program of the right and left eye, showing bilateral superior altitudinal field defects**.

**Figure 2:**
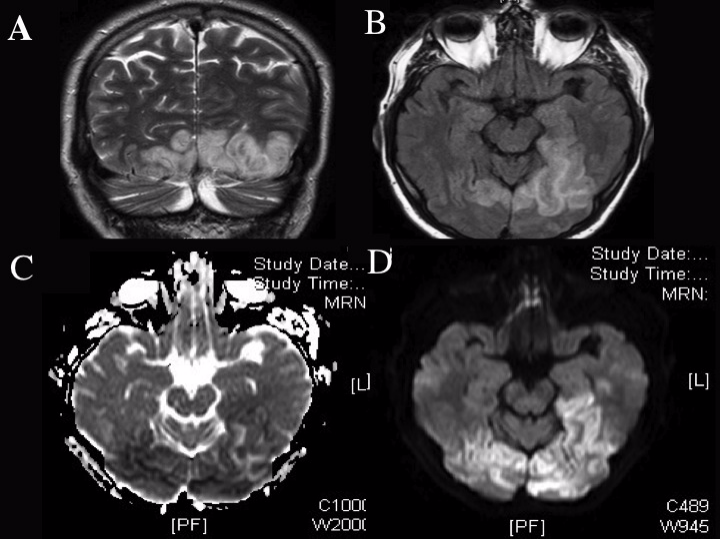
**(A) MRI scan coronal view, T2 weighted, (B) axial FLAIR sequence, (C) Acquired diffusion coefficient and (D) Diffusion weighted image, showing area of abnormal signal in both occipital lobes and extending into the inferomedial aspect of the left temporal lobe, consistent with acute cerebral infarction**.

On waking from the anaesthetic, the patient immediately complained of visual disturbance. On examination the patient had a visual acuity of 2/200 in both eyes and computerised static perimetry performed day 1 post ablation showed bilateral superior altitudinal field defects (figure 1). Full neurological examination was undertaken and in addition to the visual deficit the patient also had subtle expressive dysphasia with word finding difficulty. Magnetic resonance imaging (MRI) of the brain on day 2 post ablation showed increased T2 FLAIR signal in both occipital lobes, extending into the inferomedial aspect of the left temporal lobe (figure 2), no evidence of older infarcts were seen. A diagnosis of multifocal cerebral infarction post catheter ablation was made.

Day 3 post ablation, he developed an episode of atrial arrhythmia and was commenced on oral amiodarone. Warfarin was withheld until day 5 post ablation and then recommenced and maintained at therapeutic levels. Blood tests for biochemical abnormalities, coagulation studies and a clotting screen were within normal limits. A review one week later, showed an improvement in the visual acuity to 20/60 in the right eye and 20/80 in the left eye with a persistent bilateral superior altitudinal field defect.

One year later, visual acuity and visual field remained unchanged and the patient continued to have ongoing difficulties with word finding and reading. His atrial fibrillation, however, was now well controlled on medical therapy (warfarin, amidarone, perindopril and metoprolol).

## Discussion

Stroke after catheter ablation for atrial fibrillation is uncommon. The procedure related risk of stroke varies from study to study. A survey of 181 centres published in 2005, with a cohort of 9370 undergoing 11762 ablation procedures observed a procedure related risk of stroke of 0.28% [2]. A recent systematic review of 72 studies (approximately 16900 patients) observed a median risk of stroke of 0.9% [1]. While the risk of stroke is low it is a significant cause of mortality, being the third most common reported cause of death in a recently published large case series [4]. Capatto et al reported that 2 of the 4 deaths observed in their worldwide survey were due to massive cerebral thromboemoblism [2].

While the risk of stroke after radiofrequency cardiac ablation for atrial fibrillation is rare, bilateral stroke or multiple emboli is even more uncommon. Patient characteristics predisposing to early thromboembolic events after catheter ablation have been difficult to study due to the low occurrence of this complication. Oral et al observed that out of their 7 patients that suffered a stroke, 6 had 1 or more risk factors for stroke [5]. Conversely however, 54% of the patients who did not suffer from a thromboembolic event also had 1 or more risk factor for stroke [5]. The majority of studies have focused on anticoagulation protocols and procedural techniques when examining thromboembolic risk profiles.

Our patient did not have a high-risk profile for stroke, with only 1 risk factor identified. He was appropriately anticoagulated before and during the procedure and the procedure itself was uncomplicated. The development of stroke in our patient suggests that there may be an inherent procedure-risk of stroke that may not be completely eliminated by adequate anti-coagulation.

Higher dose heparin infusions are used intra-operatively to prevent thrombus formation. However, it has been postulated that while heparin prevents thrombus formation, it does not prevent coagulum formation [6]. Coagulum is heat-denatured fibrinogen which results from overheating at electrode surfaces and may embolise or form a nidus for thrombus formation [6]. Recent studies have suggested continuing warfarin therapy throughout the perioperative period to further reduce the risk of clot formation and thus the risk of thromboembolic stroke [7]. This, however, may still carry the risk of a shower of thromboemboli resulting in multiple cerebrovascular infarctions as demonstrated in our case.

## Conclusion

While the risk of thromboembolism and perioperative stroke during radiofrequency catheter ablation is small, it is not insignificant. To the best of our knowledge, this is the first case report of a bilateral altitudinal field defects secondary to bilateral occipital lobe infarction following radiofrequency cardiac catheter ablation. Further studies examining risk factors that may put patients at higher risk of stroke with catheter ablation need to be undertaken so that therapeutic strategies can be targeted towards these patients and reduce their risk of debilitating stroke.

## Consent

Written informed consent was obtained from the patient for publication of this case report.

## Competing interests

The authors declare that they have no competing interests.

## Authors' contributions

STL carried out preparation and drafted the manuscript. CSC and AWL were the treating doctors involved in the investigation and management of the case. All authors read and approved the final manuscript.

## Pre-publication history

The pre-publication history for this paper can be accessed here:

http://www.biomedcentral.com/1471-2261/10/14/prepub
